# Evaluation of the Osteogenic Potential of a NOTCH1 Agonist and Poloxamer 407 Hydrogel Regarding Osteoblasts

**DOI:** 10.3390/biology15030217

**Published:** 2026-01-24

**Authors:** Subburaman Mohan, Chandrasekhar Kesavan

**Affiliations:** 1 Musculoskeletal Disease Center, VA Loma Linda Healthcare System, Loma Linda, CA 92357, USA; subburaman.mohan@va.gov; 2 Department of Medicine, Loma Linda University, Loma Linda, CA 92354, USA; 3 Orthopedic Surgery, Loma Linda University, Loma Linda, CA 92354, USA

**Keywords:** P407, osteoblasts, Yhhu3792, scaffold

## Abstract

In this study, the osteoinductive activity of a small-molecule NOTCH1 activator, Yhhu3792, and Poloxamer 407, an FDA-approved hydrogel, was evaluated independently regarding osteoblast functions in vitro using primary cultures of osteoblasts derived from C57BL/6J mice. We found that treatment with Yhhu3792 increased the number of NOTCH1-positive osteoblasts (36%) compared to the vehicle control (19%) after antibody staining, suggesting increased NOTCH1 signaling after Yhhu3792 treatment. Osteoblasts treated with varying doses (5, 10, and 20 μM) of Yhhu3792 and P407 (1–25%) stimulated both osteoblast proliferation and differentiation by 25–45% (*p* < 0.05) compared to the vehicle control. Accordingly, 10 µM Yhhu3792 treatment for 9 days increased the alizarin red-stained mineralized nodule area (8.69 ± 0.97 vs. 4.05 ± 1.51 arbitrary units; *p* < 0.05) compared to the vehicle treatment. Similarly, osteoblasts treated with 10% P407 also significantly increased mineralized nodule formation. The Cell Tox Green dye assay revealed that the dosage of Yhhu3792 used was not cytotoxic. Gene expression studies measured by real-time PCR revealed that a 24 h treatment with 10 µM Yhhu3792 significantly increased expression levels of bone formation markers (*Vegf*, *Osteocalcin*) and NOTCH1 targets (*c-myc*, *Cox2*, and *Hes1)* in osteoblasts. A low dose of P407 in combination with 10 µM Yhhu3792 stimulated a significant increase (>40%) in the proliferation of bone marrow stromal cells. In conclusion, our in vitro findings showing osteogenic effects of the small molecule Yhhu3792 and P407 hydrogel should be confirmed in vivo in animal fracture healing models.

## Simple Summary

To facilitate healing of bone injuries, there is a need for small-molecule therapeutics and bone substitutes that are osteogenic, non-toxic, and capable of releasing therapeutics. In this study, we assessed the importance of the NOTCH1 agonist Yhhu3792 and the Poloxamer 407 (P407) hydrogel on bone-forming osteoblasts and bone marrow stromal cells. Our findings indicate that Yhhu3792 has an anabolic effect on osteoblast proliferation and differentiation, promoting mineralization with little or no toxicity. P407 exhibits osteogenic effects at low doses, and the combination of Yhhu3792 with P407 significantly enhances bone marrow stroma cell functions compared to treatment with either agent alone. Therefore, our in vitro findings demonstrating the osteogenic effects of Yhhu3792 and P407 hydrogel warrant confirmation in vivo in animal fracture healing models.

## 1. Introduction

Non-union bone defects, occurring because of injuries, lifestyle factors, and co-morbidities, generate high costs and can lead to serious disabilities [1,2,3,4]. Therefore, developing strategies to reduce the occurrence of non-union fracture formation and/or identifying treatments for critical-size bone defect repair, regardless of co-morbidities [3], could significantly improve the quality of life during the bone repair process and lower healthcare costs. The healing of fractured bone in part mimics the endochondral bone formation process, which is induced by multiple receptor-mediated signaling pathways. Identifying these anabolic signaling pathways and assessing their effects on bone healing using small-molecule agonists could have a substantial translational impact. In this study, we focused on the neurogenic locus NOTCH homolog protein 1 (NOTCH1) anabolic signaling pathway for the following reasons: (1) NOTCH signaling is upregulated throughout all phases of fracture healing (endochondral and intramembranous bone formation), indicating the importance of NOTCH signaling in bone repair [5]. Studies have shown that over-expression of NOTCH signaling accelerated fracture healing and that removal of NOTCH signaling resulted in increased non-union healing and reduced fracture callus stiffness in mice [6,7]. (2) NOTCH1 signaling is required for differentiation of skeletal stem cells into osteoblast-lineage cells [8,9]. (3) Inhibition of NOTCH signaling in the αSMA lineage decreased osteoblast progenitors, reduced vascularization, and sustained inflammation 10 days post-injury and enhanced inflammation 42 days post-injury [8]. These data from independent studies demonstrate that NOTCH1 signaling is important for the healing of fractured bone.

A recent study identified a small-molecule compound, N2-(4-isopropylphenyl) 5-(3-methoxyphenoxy) quinazoline-2,4-diamine, also known as Yhhu-3792, which activates NOTCH1 signaling [10]. The findings of this study revealed that treatment with Yhhu3792 increased NOTCH1 target expression, cell survival, proliferation, and neuronal stem cell function both in vitro and in vivo [10]. Additionally, blocking NOTCH1 expression abolished Yhhu3792 effects, suggesting the NOTCH1-dependence of Yhhu3792 because of its biological effects [10]. Although NOTCH1 is well documented in bone healing, the effect of Yhhu3792 on osteoblasts remains unexplored.

Currently, to deliver bone anabolic therapeutics at a bone defect site, a wide variety of biodegradable scaffolds in combination with synthetic substances have been tested [11,12] and have shown variable effects in bone healing applications. Currently, tricalcium phosphate (TCP), an osteogenic biomaterial, is widely used for bone surgeries with internal fixation at non-union sites to promote bone healing because autografts and allografts are not readily available. However, the results have been mixed because of the rapid degradation of TCP [13] (32204008). To address this issue, TCP has been combined with other materials; however, degradation of these materials (e.g., PGLA) triggered an adverse inflammatory tissue response. Thus, there is a need to develop a scaffold with a combination of appropriate materials that can be optimized for mechanical stability, slower degradation rates, and osteogenic potential to promote healing of non-union or critical-size bone defects. In this study, we focused on P407, a triblock copolymer consisting of a central hydrophobic block of polypropylene glycol flanked by two hydrophilic blocks of polyethylene, for the following reasons. P407 is an FDA-approved polyoxypropylene block polymer that gels when handled at room temperature and has been widely utilized for oral solutions, suspensions, and inhalation formulations. Studies indicate that P407 holds a significant promise for treating periodontal disease and serves as a support matrix for cell proliferation, differentiation, and the on-demand release of biomolecules and drugs [14,15]. Additionally, recent investigations have demonstrated that P407, when combined with other biomaterials, can sustain the viability of dental stem cells [16]. However, the osteogenic effect of P407 has not been tested. Therefore, the goal of this study was to assess the anabolic effects of Yhhu3792 and/or P407 on osteoblast functions in vitro.

## 2. Materials and Methods

### 2.1. Cell Culture

Primary osteoblasts were isolated from marrow-flushed long bones of adult male C57BL/6J mice after collagenase digestion, as described in [17]. The cells were cultured in media with serum and antibiotics at 37 °C in a 5% CO_2_ incubator.

### 2.2. Immunofluorescence Assay

Osteoblasts treated with the vehicle and 10 µM Yhhu3792 were fixed according to a cell signaling immunofluorescence protocol and incubated with anti-activated NOTCH1 antibody (Ab8925, Abcam, Boston, MA, USA). Forty-eight hours later, the cells were washed three times for 5 min with 1× phosphate-buffered saline (PBS) (chemicals purchased from St. Louis, MO, USA) containing 0.01% tween 20 and incubated with Alexa 555 fluorochrome-conjugated secondary antibody (source: Goat, Cell Signaling, Danvers, MA, USA) diluted in antibody dilution buffer for 2 h and protected from light, with gentle shaking. After 2 h the cells were rinsed with PBS once and incubated with 4′,6-diamindino-2-phenylindole (DAPI) (Cell Signaling, Danvers, MA, USA) for nuclear staining and rinsed three times for 5 min with PBS. The cells were protected from light and visualized under 50× magnification using a Lecia fluorescence microscope (Bannockburn, IL, USA).

### 2.3. Proliferation and Differentiation Assessment

Six thousand cells/well were plated in a 96-well plate for both assays and the cells were cultured in media with serum for 24 h followed by media containing 0.1% bovine serum albumin (Sigma, St. Louis, MO, USA and Fisher Scientific, Waltham, MA, USA) for 24 h and then for 48 h with varying doses of Yhhu3792, P407, or the vehicle. The Yhhu3792 was dissolved in DMSO (Sigma, St. Louis, MO, USA) and P407 in Millipore water. Proliferation was assessed using a Cy-Quant Dye Kit (Life Technologies, Danvers, MA, USA) according to the manufacturer’s instructions. The cells were cultured in media containing 0.1% bovine serum albumin (BSA) with 50 ug/mL of ascorbic acid (Sigma, St. Louis, MO, USA) and 10 mM of β-glycerophosphate (Sigma, St. Louis, MO, USA) for 72 h to assess differentiation by measurement of alkaline phosphatase (ALP) activity. After 24 hours, the ALP substrate was added to the lysis buffer (prepared in-house) and ALP activity was measured using a plate reader at an absorbance of 410–490 nm. Total protein was measured in lysis buffer using a bicinchoninic acid assay (BCA). The ALP activity was adjusted for the total cellular protein concentration and expressed as U/mg of protein [17].

### 2.4. Mineralization

Osteoblasts were cultured in differentiation media containing 10% calf serum (Thermo Fisher Scientific, Waltham, MA, USA) for 9 days with Yhhu3792 or 17 days with P407 (10%). A DMSO vehicle was used for comparison. The media was changed every 3 days, during which the Yhhu3792 or P407 was added. At the end of the treatment, the cultures were washed in PBS and fixed in 10% cold ethanol and subjected to alizarin red staining [18]. Image Fiji freeware of ImageJ2 was used to calculate the mineralized area. The images were converted into 8 bits, thresholds were adjusted to highlight the nodules, and the areas were measured.

### 2.5. Gene Expression

Osteoblasts were plated and cultured for 48 h in media with serum in 6-well plates followed by 24 h in serum-free media (Sigma, St. Louis, MO, USA and Thermo Fisher Scientific, Waltham, MA, USA). Subsequently, the cells were treated with the vehicle (DMSO) or 10 µM of Yhhu3792. Total RNA was isolated twenty-four hours after treatment with Yhhu3792. The cDNA was synthesized and subjected to real-time PCR amplification using a SYBR green master mix and gene-specific primers (IDT DNA technology, Coralville, Iowa, USA) in a ViiA7 real-time PCR system (Applied Biosystems-Thermo Fisher Scientific, Waltham, MA, USA). An endogenous control (β*-actin*) was used to normalize the data, and the normalized values were subjected to the 2^∆∆^Ct formula (where Ct is the contraction threshold) to calculate the percentage change between the vehicle and experimental groups. The gene-specific primer sequences were as follows: *Hes1*, F 5′ CTGAGCACAGAAAGTCATCAAAGCC 3′ and R 3′ GGTATTTCCCCAACACGCTCG 5′; *Cox2*, F 5′ GCCCGACACCTTCAA CATTGA A 3′ and R 3′ TCTCAATGAGTACCGGAAACG C 5′; *Oc*, F 5′ CTCTCTCTGCTCACTCTGCT and R 3′ TTTGTAGGCGGTCTTCAAGC 5″; *β-actin*, F 5′ CAG GCA TTG CTG ACA GGA TG and R 3′ TGCTGATCCACATCTGCTGG 5′; *c-myc*, F 5′ CAAGAGGCGGACACACAAC 3′ and R 3′ GGCCTTTTCGTTGTTTTCCA 5′; and *Vegf*, F 5′ ATATCAGGCTTTCTGGATTAAGGAC and R 3′ CAGACGAAAGAAAGACAGAACAAAG 3′.

### 2.6. Cell-Tox Green Cytotoxicity Assay

Cells were cultured in media with 10% serum followed by serum-free media for 24 h in the presence or absence of Yhhu3792. Twenty-four hours after incubation, CellTox Green Cytotoxicity and assay buffer (Promega, Madison, WI, USA) were prepared and 100 µL of this mixture was added to the cells with media. The plates were incubated for 15 min in the dark and the fluorescent signals were measured after a 485–500 nm excitation and at 520–530 nm. Lysis buffer (Promega, Madison, WI, USA) was added to one row of control cells and cell toxicity was measured as a positive control.

### 2.7. Statistical Analysis

The descriptive statistics were used to calculate the average and standard error of mean (SEM). One-way ANOVA and Student’s *t*-test were used to evaluate the differences between the vehicle control (DMSO) and compound-treated cultures using Microsoft Excel and Statistica software (Version 10). A *p*-value of <0.05 was considered statistically significant vs. the untreated control (cells without any treatment) or vehicle control (cells treated with DMSO) as appropriate.

## 3. Results and Discussion

**Figure 1:**
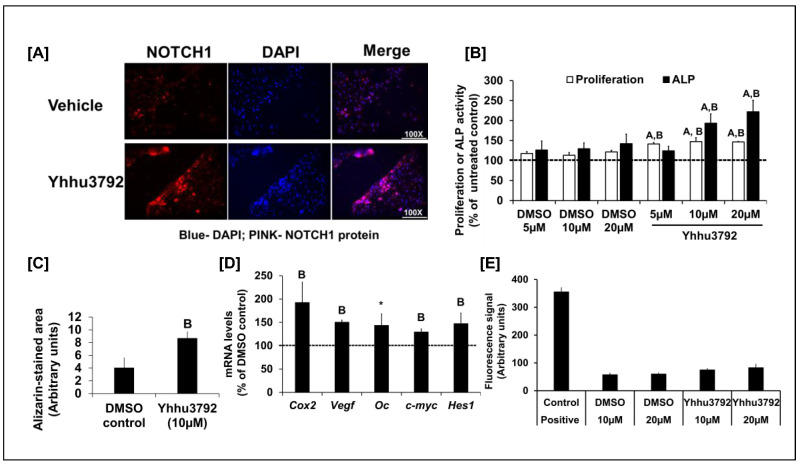
(**A**) Increased level of activated NOTCH1 in osteoblasts (OBs) treated with 10µM Yhhu3792 for 30 min by immunofluorescence assay (image taken at 100× magnification). NOTCH1 activation is shown by red fluorescence in the nuclei. Nuclei were stained blue with DAPI, a blue fluorescence; *n* = 3. (**B**) Changes in osteoblast proliferation and differentiation in response to varying doses of DMSO solvent, the control, or Yhhu3792. The values for untreated control were 324 ± 44 (arbitrary units) for proliferation and 7.8 ± 1.9 mU/mg of protein for alkaline phosphatase (ALP) activity. Values are % of the untreated control (*n* = 7–8). (**C**) Effect of 10 µM of Yhhu3792 on mineralized nodule formation (*n* = 3–4). (**D**) Changes in expression levels of NOTCH target and bone formation marker genes in osteoblasts after a 24 h treatment with 10 µM Yhhu3792 (*n* = 3–4). The delta Ct values of the DMSO control were 5.4 ± 0.18, 9.71 ± 0.13, 11.35 ± 0.21, 5.85 ± 0.10, and 10.20 ± 0.25 for *Cox2*, *Vegf*, *Oc*, *c-myc*, and *Hes1*, respectively. Values shown are % of the DMSO-treated control cultures (*n* = 4). (**E**) Measurement of cell death in the DMSO vehicle and Yhhu3792-treated osteoblasts using the Cell Tox Green DNA binding dye assay (*n* = 8). Values are the mean ± SEM. ^A^ *p* < 0.05 vs. the control (without any treatment) and ^B^ *p* < 0.05 and * *p* = 0.07 vs. the DMSO control (vehicle control) according to Student’s *t*-test. One-way ANOVA revealed *p* < 0.00001 between all groups vs. untreated groups as well as vehicle (DMSO) treatments regarding proliferation. For ALP activity, one-way ANOVA showed *p* < 0.001 between the treatment (Yhhu3792) and control groups. The dashed line reflects 100% cut off value.

Cellular and molecular processes involved in fracture healing are known to mimic events that occur during embryonic skeletal development. Among the various signaling pathways implicated in skeletal development, NOTCH signaling has received much attention based on the findings from transgenic mouse studies that implicate a key role for NOTCH signaling in bone formation during both development and fracture healing [6,7,8]. Direct activation of NOTCH receptors using recombinant NOTCH ligands is not a viable treatment strategy to increase bone formation because of the short half-life of protein therapy and the expense involved in generating recombinant proteins. Therefore, much attention has been focused on the identification and characterization of small molecular activators and inhibitors of receptors towards the development of therapeutics. A recent report [10] highlighted the utility of the small molecule Yhhu3792 in promoting the proliferation and differentiation of neuronal stem cells through NOTCH1 signaling. Given the crucial role of NOTCH1 in skeletal development, we evaluated the biological activity of the small molecule Yhhu3792, a NOTCH1 agonist, on osteoblasts. Osteoblasts were treated in culture with 10 µM Yhhu3792 or a vehicle control. After 30 min, NOTCH1 activation was monitored by immunofluorescence using an anti-activated NOTCH1 antibody, which specifically recognizes and binds to activated NOTCH1. Figure 1A demonstrates that Yhhu3792 treatment increased activated NOTCH1 levels in osteoblasts, consistently with published findings in neuronal cells and other cell types [19]. However, further in vivo studies using NOTCH1 reporter mice are necessary to validate the specificity of these in vitro findings.

To assess whether Yhhu3792 enhanced osteoblast cellular functions, osteoblasts were isolated from the long bones of 12-week-old male C57BL/6J mice and cultured in vitro. Treatment with Yhhu3792 caused a 24% (*p* < 0.05) increase in osteoblast proliferation after 48 h (Figure 1B). To evaluate the effect of Yhhu3792 on osteoblast differentiation, osteoblasts were treated with different doses of Yhhu3792 in differentiation media [17] for evaluation of ALP activity. We found that ALP activity increased by 30–40% at 10 and 20 µM doses of Yhhu3792 compared to those treated with vehicle. By contrast, no change in ALP activity was observed at 5 µM, suggesting that this dose was insufficient to increase ALP activity (Figure 1B). The lack of a dose-dependent response in proliferation or differentiation to Yhhu3792 in our study could be attributed to the narrow range of doses tested. A broader dose–response study is needed to determine the optimal dose of Yhhu3792 required to stimulate osteoblast function. To further support the finding that Yhhu3792 enhances osteoblast differentiation, we assessed its effect on mineralization in vitro using alizarin red staining (Figure 1C). We observed increased mineralization, as indicated by alizarin red staining, in osteoblasts cultured for 8 days in the presence of 10 µM Yhhu3792 in differentiation media. Semi-quantitative analysis revealed that the alizarin red-stained mineralized area was increased more than 2-fold in Yhhu3792-treated cultures compared to the DMSO control (Figure 1C).

To evaluate if the effect of the small molecule Yhhu3792 on osteoblasts was mediated through activation of NOTCH1 signaling, we measured expression levels of NOTCH1 targets after a 24 h treatment with 10 µM Yhhu3792 in serum-free media. We found that the expression levels of bone formation markers (*Vegf* and *osteocalcin*) and NOTCH1 targets (*c-myc*, *Cox2*, and *Hes1*) were increased in response to Yhhu3792 treatment (Figure 1D). Previous research has demonstrated that Yhhu3792 is not toxic to neuronal cells in vitro and in vivo [10]. To confirm this finding in osteoblasts, we next determined if Yhhu3792 exerted effects on osteoblast apoptosis. Using the CellTox Green Cytotoxicity assay, we found that Yhhu3792 at the doses tested was not cytotoxic compared to corresponding vehicle treated cultures. By contrast, the positive control exhibited a significant increase in apoptosis, as expected (Figure 1E).

**Figure 2:**
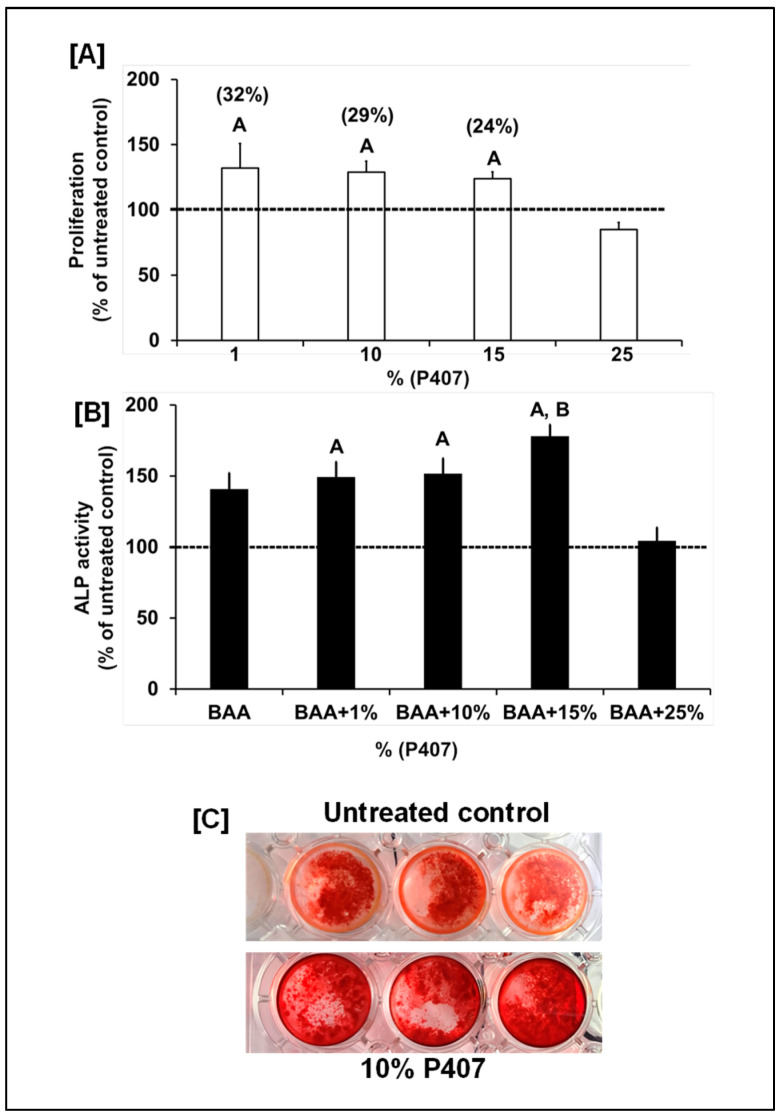
(**A**) Changes in osteoblast number in response to varying doses of P407. Values are % of the untreated control (*n* = 8). The values for the untreated control were 234 ± 17 (arbitrary units) for proliferation. (**B**) Changes in osteoblast differentiation as reflected by alkaline phosphatase (ALP) activity after 72 h in response to varying doses of P407 in differentiation media. Values are % of the untreated control (*n* = 6–8). The control values for ALP activity were 2.0 ± 0.27 mU/mg of protein. (**C**) P407’s effects on formation of alizarin red-stained mineralized nodules (*n* = 3). ^A^ *p* < 0.05 vs. the untreated control (no BAA) and ^B^ *p* < 0.05 vs. β-glycerophosphate and ascorbic acid (BAA) according to the Student’s *t*-test. One-way ANOVA showed *p* < 0.0001 between the treatment (BAA and P407) vs. untreated control groups for both proliferation and ALP activity.

Presently, to deliver bone anabolic agents or small-molecule therapeutics into a nonunion bone fracture, a wide variety of biodegradable ceramics have been tested together with internal fixation to promote bone healing because autografts and allografts are not readily available. In this study, we focused on P407 based scaffolds because a recent study showed that P407 improved injectability and shape stability of beta-tricalcium phosphate (TCP) cement [20]. Another study showed that the addition of silicon oxide to a P407 hydrogel-based hydroxyapatite bone substitute improved its mechanical stability without affecting the in vivo healing capacity of P407-hydroxyapatite bone substitute material [19]. To determine if P407 exerts osteoinductive effects on osteoblasts, we evaluated the biological effects of P407 on osteoblast proliferation and differentiation. We found that P407 at doses between 1, 10 and 15% but not 25% increased osteoblast proliferation and differentiation (Figure 2A,B). Dose response studies on the effects of P407 on osteoblast differentiation revealed that P407 only at a dose of 15% significantly increased ALP activity compared to β-glycerophosphate and ascorbic acid (BAA) treated control cultures (Figure 2B). To further substantiate the finding that P407 increased osteoblast differentiation, the effect of Yhhu392 and P407 on mineralization, was assessed in vitro using alizarine red staining. We found that osteoblasts cultured with 10% P407 in the differentiation media containing 10% calf serum and BAA for 17 days showed increased mineralization as reflected by alizarin red staining (Figure 2C). Our study provides the first in vitro evidence that P407 is osteogenic.

**Figure 3:**
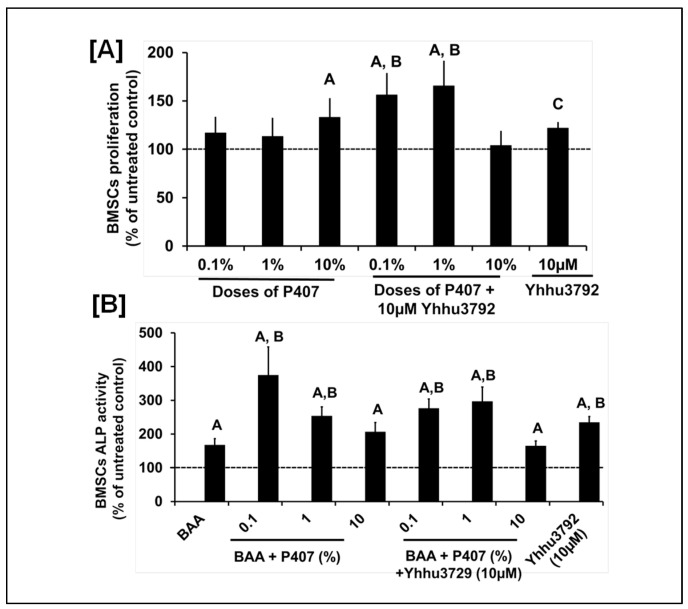
Changes in BMSC proliferation (**A**) and ALP activity (**B**) in response to treatment with Yhhu3792 or P407 alone or in combination. Values are the mean ± SEM (*n* = 6–8) For proliferation, ^A^ *p* < 0.05 vs. the untreated control and ^B^ *p* < 0.05 vs. varying doses of P407 and ^C^ *p* < 0.05 vs. the untreated control. For ALP activity, ^A^ *p* < 0.05 vs. the untreated control BMSCs (without BAA) and ^B^ *p* < 0.05 vs. BMSCs with BAA according to the Student’s *t*-test. The values for the untreated control were 232 ± 22 (arbitrary units) for proliferation and 9.8 ± 2.72 mU/mg of protein for ALP activity differentiation, reflected by an increase in ALP activity (Figure 3B). We also found that the combination of P407 and Yhhu3792 signficantly increased ALP acitivity compared to vehicle treatment, however, the combined effect was not greater compared to that of each individual treatment.

It is well known that scaffold materials contain chemical structures that can alter drug structures through interactions, thereby influencing various behaviors such as stability, absorption, encapsulation, and toxicity. P407 has a free hydroxyl group (OH) and a hydrogen atom (H) on either side of the structure while Yhhu3792 contains flanking oxygen and amine (NH_2_) groups. To determine if interactions between Yhhu3792 and P407 negatively affect the osteogenic effects of Yhhu3792 or P407, we evaluated the biological effects of P407 and Yhhu3792 either alone or in combination using bone marrow stem cells (BMSCs) that promote bone healing at the injury site. We found that treatment with P407 at 10% and Yhhu3792 at 10 µM significantly increased proliferation compared to the vehicle control. Furthermore, P407 at doses of 0.1% and 1% when combined with 10 µM of Yhhu3792 resulted in a significant increase (>40%) in BMSC proliferation compared to either P407 or Yhhu3792 alone or to the vehicle control (Figure 3A). Additionally, we assessed if the Yhhu3792-P407 combination also promoted osteoblast differentiation. We found that 0.1% and 1% doses of P407 and Yhhu3792, respectively, increased osteoblasts differentiation.

The limitations of this study are as follows: (1) NOTCH signaling has been shown to vary depending on the gender. In this study, we primarily used osteoblasts and BMSCs derived from male mice. Additional studies utilizng osteoblasts and BMSCs derived from female mice are essential to ascetain whether the anabolic effects of Yhhu3792 and P407 are similar in the two genders or different. (2) We utilized osteoblasts and BMSCs derived from adult male mice to evaluate the effects of Yhhu3792 and P407 in this study. Additional studies involving aged mice are essential to determine if Yhhu3792 andP407 rescue defective osteoblast function caused by age. (3) The conclusions drawn from this study are based on in vitro models. In vivo animal studies using appropriate bone healing models and different doses of P407 and Yhhu3792 are needed to identify the doses of P407 and Yhhu3792 that are optimal to promote healing of nonunion fractures and to assess whether this bone healing occurs through NOTCH1 signaling.

In summary, our findings show that (1) the small molecule Yhhu3792 acts through NOTCH1 signaling in osteoblasts to stimulate osteoblast cellular functions, (2) a low dose of P407 is osteogenic, and (3) P407 in combination with Yhhu3792 significantly increased BMSC proliferation but not ALP activity when compared to P407 or Yhhu3792 alone.

## 4. Conclusions

Our in vitro findings demonstrate the osteogenic effect of a small-molecule NOTCH1 agonist, Yhhu3792, and the P407 hydrogel. The utility of this therapeutic should next be confirmed in vivo in animal fracture healing models.

## Data Availability

Raw data are available on request.
